# Microchip Screening Platform for Single Cell Assessment of NK Cell Cytotoxicity

**DOI:** 10.3389/fimmu.2016.00119

**Published:** 2016-04-05

**Authors:** Karolin Guldevall, Ludwig Brandt, Elin Forslund, Karl Olofsson, Thomas W. Frisk, Per E. Olofsson, Karin Gustafsson, Otto Manneberg, Bruno Vanherberghen, Hjalmar Brismar, Klas Kärre, Michael Uhlin, Björn Önfelt

**Affiliations:** ^1^Science for Life Laboratory, Department of Applied Physics, KTH – Royal Institute of Technology, Solna, Sweden; ^2^Department of Microbiology, Tumor and Cell Biology, Karolinska Institutet, Stockholm, Sweden; ^3^Center for Allogeneic Stem Cell Transplantation, Huddinge University Hospital, Karolinska Institute, Stockholm, Sweden; ^4^Department of Oncology and Pathology, Karolinska Institutet, Stockholm, Sweden

**Keywords:** NK cells, cytotoxicity, single cell analysis, microchip, screening, microscopy, fluorescence, immune synapse

## Abstract

Here, we report a screening platform for assessment of the cytotoxic potential of individual natural killer (NK) cells within larger populations. Human primary NK cells were distributed across a silicon–glass microchip containing 32,400 individual microwells loaded with target cells. Through fluorescence screening and automated image analysis, the numbers of NK and live or dead target cells in each well could be assessed at different time points after initial mixing. Cytotoxicity was also studied by time-lapse live-cell imaging in microwells quantifying the killing potential of individual NK cells. Although most resting NK cells (≈75%) were non-cytotoxic against the leukemia cell line K562, some NK cells were able to kill several (≥3) target cells within the 12-h long experiment. In addition, the screening approach was adapted to increase the chance to find and evaluate serial killing NK cells. Even if the cytotoxic potential varied between donors, it was evident that a small fraction of highly cytotoxic NK cells were responsible for a substantial portion of the killing. We demonstrate multiple assays where our platform can be used to enumerate and characterize cytotoxic cells, such as NK or T cells. This approach could find use in clinical applications, e.g., in the selection of donors for stem cell transplantation or generation of highly specific and cytotoxic cells for adoptive immunotherapy.

## Introduction

Cytotoxic effector lymphocytes, such as natural killer (NK) cells and T cells, are important for immune defense against cancer and viral infections, the traits that have made these cells valuable in adoptive cell therapy. However, their activity is also associated with detrimental conditions, such as autoimmunity or graft-versus-host disease (GVHD), after allogeneic hematopoietic stem cell transplantation (HSCT). Upon activation, both effector cell types are able to kill abnormal cells through release of toxic granules containing perforin and granzymes at the tight intercellular contact formed at the immune synapse (1, 2).

NK cell activation relies on a balance between activating and inhibitory signals from a range of cell surface receptors recognizing ligands on the target cell surface. Inhibitory signals are mediated by MHC class I proteins that are expressed by most normal cells. However, some infections and transformations lead to downregulation of MHC class I and/or upregulation of activating NK cell ligands rendering them susceptible to NK cell attack. A functional NK cell repertoire is generated through cellular education, resulting in a heterogeneous NK cell population with varying capacity to respond to stimuli (3–6). Little is known about the functional consequences of education and how this relates to the individual NK cell cytotoxic response observed. However, clinical trials using NK cells from haploidentical donors for cell therapy have shown encouraging results indicating that interindividual differences in NK cell recognition and responsiveness can be used to treat disease (7). Importantly, these studies also established a link between the number of alloreactive NK cells in the graft and patient survival. However, one limitation is that there are few efficient methods to enumerate the fraction of cytotoxic NK cells from a donor sample for a given donor–recipient pair. Thus, new methods to quantify the fraction of alloreactive NK cells and cytolytic potential of individual NK cells could be valuable for the process of selecting donors for therapy.

During the past years, several new tools for single cell analysis have been developed, and some of those have been used to dissect T or NK cell heterogeneity in terms of phenotype, cytotoxicity, or cytokine release (8–22). Here, we use a previously reported microchip platform (23, 24) to screen the cytotoxic response of human peripheral blood NK cells against transformed human cells. This tool complements currently used population- and flow-based techniques as it quantifies the fraction of cytotoxic cells and resolves the cytotoxic potential of individual cells. We find donor-to-donor differences in the fractions of cytotoxic NK cells, a dependence on the choice of target cell and significant heterogeneity in cytotoxic capacity of individual cells.

## Materials and Methods

### Microchip and Holder

Fabrication of microchips was performed as previously described (24). Briefly, microwell layout was defined by lithography followed by deep-reactive ion etching and surface oxidation growth. The microwells were sealed at one end by anodic bonding of a thin (175 μm) glass to the silicon, and the wafer was diced into individual microchips (22 mm × 22 mm × 475 μm). During loading and imaging, the microchip was placed in a custom-made holder with plastic lid held together by four magnets. To reduce evaporation, but maintain oxygen and carbon dioxide exchange, a 35-mm Petri dish lid was placed over the holder lid. Before cell seeding, the chip was covered with cell medium and primed in vacuum to allow liquid to enter the wells.

### Cells and Reagents

All experiments with human cells were performed according to local ethics regulations. Human NK cells were isolated from PBMCs of anonymous healthy donors by negative selection using the NK cell Isolation Kit (Miltenyi Biotec) according to manufacturer’s instructions. Freshly isolated NK cells were maintained in RPMI 1640 cell culture media (Sigma-Aldrich) supplemented with 10% human serum (Blood bank, Karolinska Hospital), 50 U/mL penicillin–streptomycin, 2 mM l-glutamine, 1× non-essential amino acids, 1 mM sodium pyruvate (all from Sigma-Aldrich), and naive NK cells were used within 2 days. For the activation of NK cells, 200 U/mL of IL-2 (Peprotech) was added to the cell culture media, and the NK cells were then used within 6–9 days. The NK cell purity of the isolated cell population was >95% CD3^−^CD56^+^ as confirmed by flow cytometry. The leukemia cell line K562 and human embryonic kidney (HEK)293T cells were cultured in RPMI 1640 media supplemented with 10% FBS, 50 U/mL penicillin–streptomycin, and 2 mM l-glutamine (all from Sigma-Aldrich).

For fluorescent staining before imaging, cells were washed, incubated with the appropriate dye dissolved in RPMI 1640 for 10 min at 37°C, washed, and used for experiments. Final staining concentrations were 0.5–1.0 μM for CellTrace Calcein Green AM, 5 μM FarRed DDAO-SE (target cells), and 0.4–0.6 μM CellTrace Calcein Red-Orange AM (NK cells) (all dyes were from Invitrogen). The family of CellTrace calcein dyes freely diffuses over the cell membrane. Once inside the cell, cytoplasmic enzymes hydrolyze the dye, causing the polarized product to leak out much more slowly than it entered. Calcein dyes give a uniform fluorescent cytoplasmic staining as long as the membrane is intact making them suitable for detection of cell viability. On the other hand, DDAO forms covalent bonds to primary amines present everywhere in the cell and remains detectable after cell death has occurred.

### Microscopy and Image Analysis

Images were obtained at 10× magnification using any of four inverted confocal microscopes (Olympus IX81, Zeiss LSM 510 Meta, Zeiss LSM 780, or Zeiss LSM 880) equipped with environmental chambers maintained at 37°C, 5% CO_2_, and motorized stages enabling automatic screening. Screening the whole chip required acquisition of 400 separate images, taking ~45 min. Often, analysis was performed only on parts of the chip.

The number of effector cells, as well as live and dead target cells in each well, was quantified either automatically by a software routine developed in Matlab or by a combination of automatic and manual analysis. Briefly, the image analysis software first identified the microwells using the transilluminated channel. Thresholds were applied to the fluorescence channels. Remaining objects were convolved with a Gaussian chosen to be of approximately the same size as the cells to improve circularity and to simplify separation of clustered cells. An algorithm based on the circular Hough transform (25) was applied to search for roughly circular objects. NK cells, due to their irregular non-circular morphology, were instead identified using an algorithm for finding connected components corresponding to single NK cells (25). The classification of live and dead target cells was performed by comparing the green (calcein green) and red (DDAO) fluorescent intensity from each individual target cell. The threshold was set either manually or automatically by selecting the threshold that maximizes the mean of the pairwise Euclidean distances between the intensity values of live and dead targets cells. The software then returned a numbered map of the detected wells and the number of objects in each well, together with a heuristically chosen figure of goodness describing the chances that the software had made a correct determination of the number and status of cells. This figure was based on several factors, such as large numbers of cells in a single well, the existence of large contiguous objects (likely to be multiple cells), bright objects that could not be identified as cells, and amount of overlap of identified cells. Accuracy of the automatic counting was decreased at screens performed at later times due to decreased fluorescence intensity (mainly affecting the NK cells) and debris from dying target cells in some of the wells. In such cases, manual counting was performed.

### Statistical Analysis

Yates Chi-square test was used to evaluate significance. *p*-Values above 0.05 were considered not significant (n.s.), whereas *p*-values below 0.05 were considered significant and marked by stars (0.01 < *p* < 0.05 marked as *, 0.001 ≤ *p* ≤ 0.01 marked as **, and *p* < 0.001 marked as ***).

## Results and Discussion

### Microchip Cytotoxicity Assay

**Figure 1:**
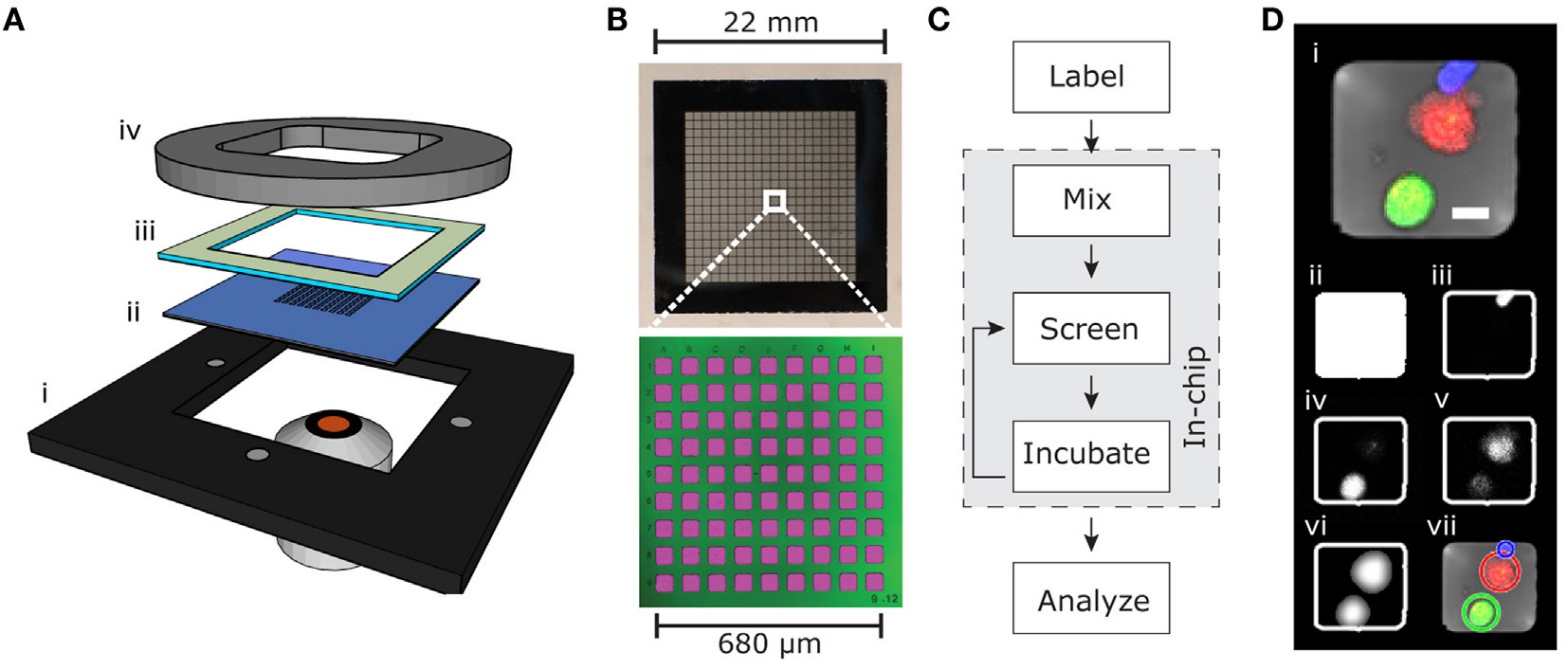
**Microchip screening platform**. **(A)** Holder (i), microchip (ii), PDMS gasket (iii), and plastic lid (iv). **(B)** Detailed view of the silicon–glass microchip showing photograph of the whole chip (top) containing 32,400 microwells arranged in 20 × 20 subunits each containing 9 × 9 microwells (bottom). The bottom image is produced by confocal fluorescence imaging of microwells filled with fluorescent solution (pink) and reflection from the silicon–glass interface (green). **(C)** Flow chart outlining the screening process. **(D)** Example RGB-transmitted light composite image (i) from an individual well containing one live target cell (green), one dead target cell (red), and one NK cell (blue). Scale bar represent 10 μm. The automated software-detected microwell is shown in (ii). The blue channel (iii) shows the calcein orange-stained NK cell, and the green channel (iv) shows the calcein green-stained target cell. The red channel (v) contains both weak DDAO fluorescence from the live target cell and stronger DDAO fluorescence from the dead target cell. The filtered image of the target cells (vi) is used for the automated software detection of live and dead target cells shown as circles in (vii).

The microchip platform consisted of a silicon–glass microchip held in place by an aluminum plate, a polydimethylsiloxane (PDMS) gasket, and a plastic lid, and it was designed to fit in a conventional inverted fluorescence or confocal microscope (Figure 1A). The microchip contained 32,400 individual wells (≈50 μm × 50 μm) arrayed to facilitate screening with a 10× objective (Figure 1B). Deep wells (300 μm) prevented cells from escaping the wells, and thin glass at the bottom (175 μm) allowed high-resolution imaging (18, 24).

To assess single cell cytotoxicity, NK cells were labeled with the fluorescent viability dye calcein orange and target cells with calcein green and DDAO. This allowed detection of live NK cells and distinguishing live from dead target cells (Figures 1C,D). Target cells were seeded on the chip and allowed to sediment in to the microwells, then NK cells were added creating an E:T ratio of ≈1:2.5–5, with the higher E:T ratio used to screen for serial killing NK cells. The chip was then screened to assess the number of effector cells and live/dead target cells in each well at the beginning of the experiment. In addition, in experiments requiring more precise enumeration of NK cell-mediated killing events, a prescreen of seeded target cells was acquired before seeding the effector cells. The cells were then left to incubate under physiological conditions (typically for 6 h) to allow effector-mediated lysis to occur before the chip was screened again. The incubation-screening cycle was repeated until the experiment was stopped. The number of effector cells and live/dead target cells was counted with the in-house automatic counting routine, and the cytotoxic potential of effector cells was assessed.

### Screening of NK-Mediated Killing of Tumor Cells

**Figure 2:**
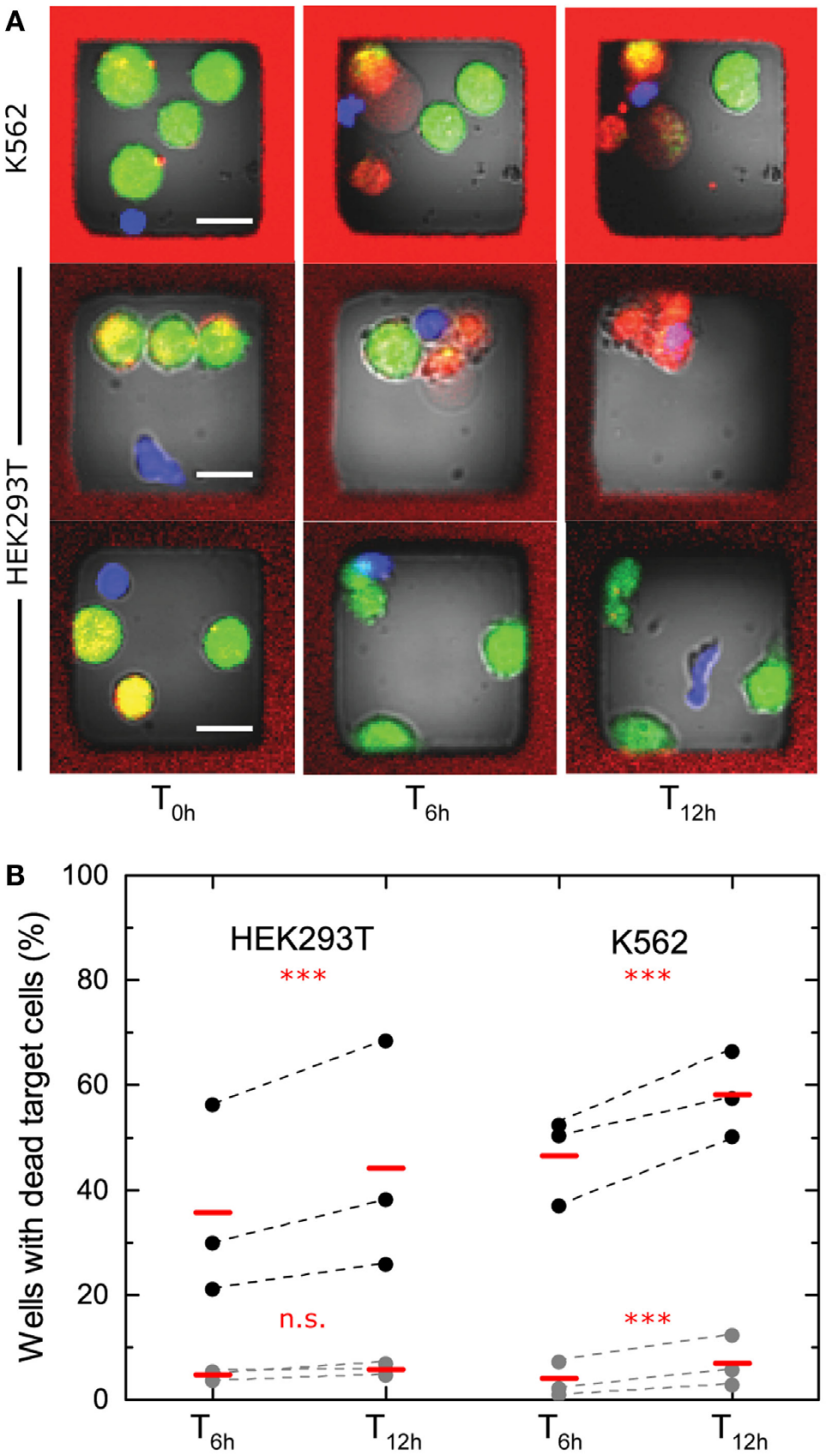
**Cytotoxicity screening of IL-2-activated human peripheral blood NK cells**. **(A)** Examples of images acquired at times 0, 6, and 12 h of wells containing single NK cell and three to four K562 (top) or HEK293T (bottom) target cells. Target cell death was observed by loss of green fluorescence from calcein and increased red fluorescence from DDAO. The NK cells shown in the top two rows both kill three target cells during the assay, whereas the NK cell shown at the bottom row is non-cytolytic. Scale bars represent 15 μm. Images have been resampled and adjusted for brightness and contrast. **(B)** Fraction of cytotoxic NK cells assessed after 6 and 12 h with HEK293T target cells (left, three separate NK cell donors, *n*_total_ = 997) or K562 target cells (right, three separate NK cell donors, *n*_total_ = 1808). Spontaneous target cell death is shown in gray (*n*_total, HEK293T_ = 2423, *n*_total, K562_ = 3467). Data points from individual donors are connected by dashed lines and mean values are shown with red bars. The presented data are based on target cells dying during the assay, i.e., death events scored between the screens at T_0h_, T_6h_, and T_12h_. Statistical analysis was performed on total number of observations for each cell type and significance is indicated by red stars or letters.

To investigate NK-mediated killing in the microchip, human peripheral blood NK cells were isolated from healthy donors and activated in IL-2 for 6–9 days. On the day of experiment, NK cells were labeled and mixed on the chip with labeled target cells. Target cells used were either the cell line K562 originally derived from a human patient chronic myeloid leukemia (CML) in blast crisis (26) or HEK293T cells. Cytolytic and non-cytolytic NK cells were observed against both types of target cells (examples in Figure 2A). To assess the level of NK killing, wells containing single NK cells and at least one live target cell at time T_0h_ were analyzed after 6 (T_6h_) and 12 h (T_12h_), and the number of wells where target cell death had occurred was counted. NK cells from three donors were tested against both types of target cell. Target cell death was observed in ≈20–70% of wells for HEK293T and in ≈30–70% of wells for K562. Spontaneous target cell death was detected in only a few percent of the wells (Figure 2B). We did note a background level of target cell death seen as a fraction (~5–7%) of the target cells being dead already at T_0h_ (Figure S1A in Supplementary Material). This fraction was higher in wells containing NK cells (≈10–15%) (Figure S1B in Supplementary Material), due to NK cell-mediated killing occurring before the first imaging was completed at T_0h_. As a consequence, the fraction of cytolytic NK cells presented in Figure 2B is slightly underestimated. The data showed that the fraction of cytolytic NK cells was somewhat higher for the K562 target cells and that the donor-to-donor differences seemed to be more pronounced for HEK293T cells. The fractions of cytolytic NK cells increased marginally between T_6h_ and T_12h_, indicating that not all lysis was completed during the first part of the assay (Figure 2B). Together, these experiments show that this screening approach allows the fraction of lytic effector cells within populations to be determined, differences between donors or types of target cells to be detected, and the change of NK cell-mediated lysis to be monitored over time.

### Time-Lapse Imaging of NK Cell Immune Surveillance

**Figure 3:**
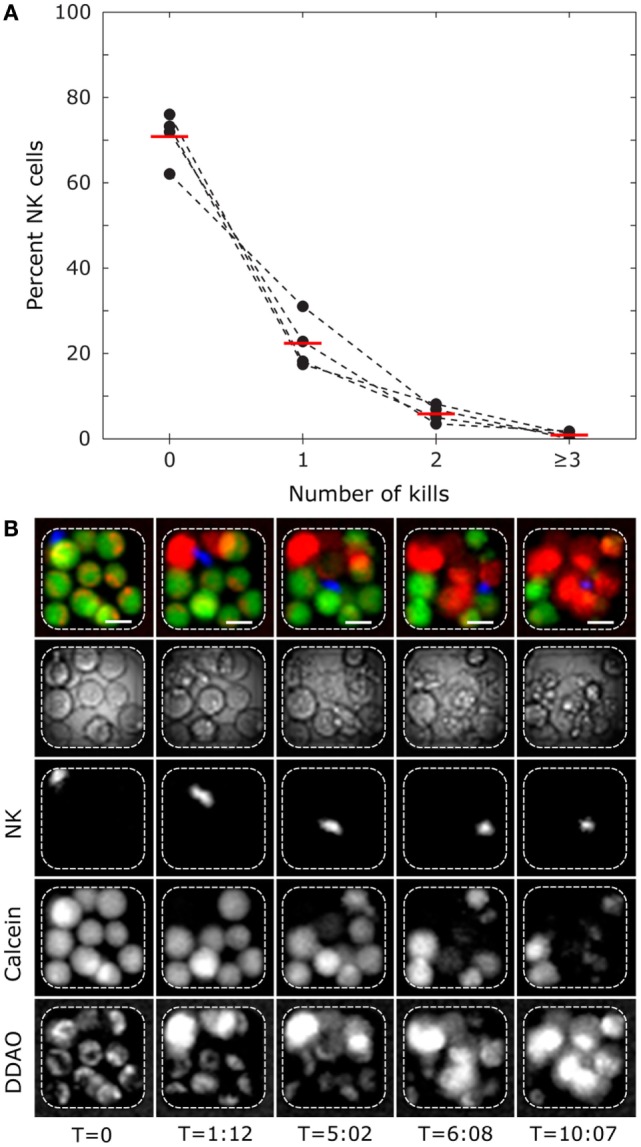
**Single-cell level cytotoxicity of resting human polyclonal NK cells revealed by time-lapse imaging in microwells**. **(A)** Frequency of NK cells killing between 0 and ≥3 target cells. Wells containing single NK cells and at least three live K562 target cells were selected for analysis. Presented data are from four donors (black circles connected by dashed lines) with mean values shown by red bars (*n*_total_ = 322). **(B)** Time-lapse sequence showing a serial killer that eliminates 8 target cells in the 12-h experiment. The rows show from top to bottom: RGB composite of fluorescent channels for NK (blue) and target cells (green and red), transmitted light, calcein orange NK channel, target cell calcein green, and target cell DDAO channels. Each column represents different time points (hours:minutes). Scale bars represent 15 μm. Images have been resampled and adjusted for brightness and contrast.

Time-lapse imaging of human NK cells interacting with K562 cells in microwells was also performed. K562 target cells followed by resting (non-activated) NK cells were seeded across the microchip at an E:T ratio of ≈1:3 and imaged at 10 parallel positions, each covering 81 microwells, every 3 min for 12 h (examples of time-lapse sequences can be found in Movies S1–S3 in Supplementary Material). From three independent experiments, 1295 NK cells and 3323 target cells distributed in 775 microwells were analyzed based on the criteria that only microwells containing at least 1 NK and 1 target cell were studied. We scored 425 killing events committed by 326 NK cells, indicating that ≈25% of the NK cells exhibited cytotoxicity. As expected, this number is lower than what was found for IL-2-activated NK cells (Figure 2). In wells with single NK cells and ≥3 live target cells, 72% of the NK cells did not kill any target cells, whereas the remaining cells were cytotoxic (Figure 3A). A small fraction of NK cells (≈1%) was scored as “serial killers,” as they killed three or more target cells during the assay. One NK cell was observed to kill as many as eight target cells (Figure 3B; Movie S3 in Supplementary Material). Although serial killers have been described before among IL-2-activated human peripheral blood NK cells (16, 17, 27), the phenomenon has been less studied among resting NK cells. We currently know very little about these NK cells with extraordinary lytic potential, partly because they are “invisible” in population-based methods or in flow cytometry where high cell surface expression of CD107a^+^ is used as a sign for degranulation and cytotoxicity after stimulation with target cells. Given that the fraction of serial killers under steady-state conditions is low, efficient methods for studying the response of single NK cells are needed to gain better understanding of these cells.

### Assessing Serial Killing by NK Cells

Finally, we decided to further evaluate the cytolytic potential of individual NK cells by adapting the assay toward accurate enumeration of killing events and detection of NK serial killers. The screening assay was adapted in three ways. (1) In the first cytotoxicity screening, it was observed that some NK cells killed targets before imaging at T_0h_ (Figure S1B in Supplementary Material); therefore, a prescreen of the seeded K562 cells were acquired prior to addition of the NK cells (T_-NK_). (2) The seeding density of target cells was doubled in order to give most NK cells a chance to kill multiple target cells. (3) Only wells that contained ≥5 live target cells at T_-NK_ and a single live NK cell were selected for further analysis.

**Figure 4:**
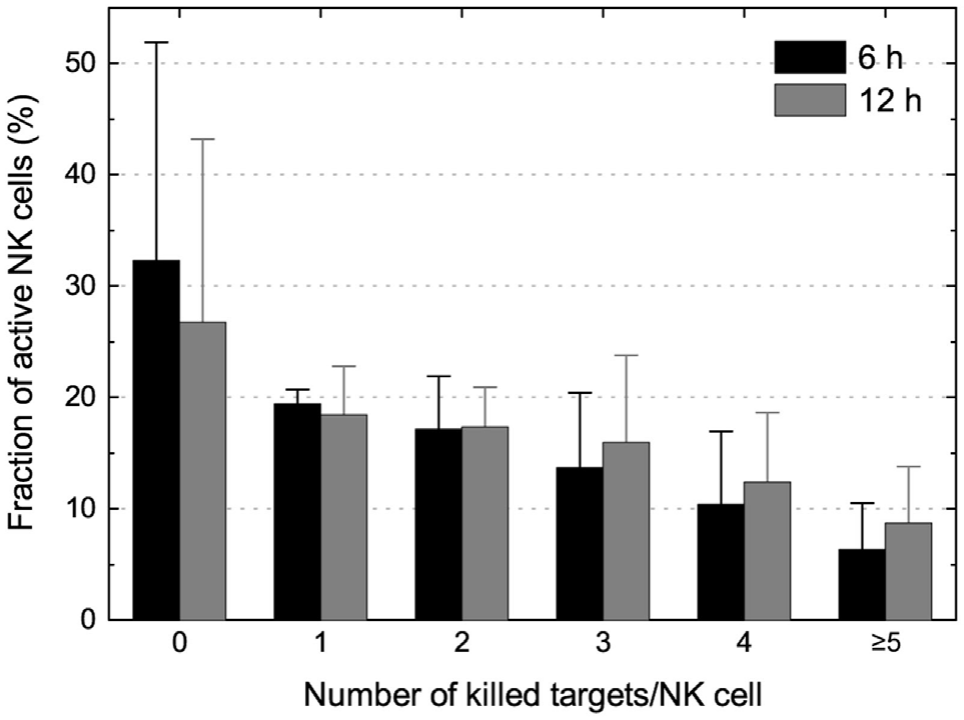
**Enumeration of killing potential and assessment of NK serial killing**. Distribution of NK cells killing 0, 1, 2, 3, 4, or ≥5 K562 target cells at T_6h_ (black bars) and T_12h_ (gray bars). Wells were selected for analysis based on having a single live NK cell at T_0h_ (and ≤1 live NK cell at later time points) and ≥5 live target cells before NK cell seeding (T_-NK_).

Pooling data from three individual experiments of IL-2-activated NK cells and K562 target cells showed that the fraction of NK cells gradually decreased with the number of killing events scored (Figure 4). Comparing the screens performed at T_6h_ and at T_12h_ showed that the majority of killing took place during the first 6 h of the assay. However, as evident from the decreased population of NK cells killing zero target cells when comparing T_6h_ and T_12h_, a small fraction of the NK cells did not perform their first kill until after 6 h. This could be caused by the requirement of initial NK–target cell interactions before activation and killing (28), but a contribution from spontaneous target cell death cannot be excluded. Interestingly, higher fractions of cytotoxic NK cells were observed in the experiments where the target cell density was higher. This is consistent with our previous report that high target cell density can increase the likelihood of NK cell cytotoxicity but could also be coupled to heterogeneity in target cells susceptibility or motility of the NK cell (16, 29).

Taken together, NK cells that killed three or more target cells were responsible for 70% of the killing, and from this cohort, the most potent NKs (killing ≥5 targets) were responsible for 20–25% of the total target cell death. Thus, despite representing a minority of the total population, the serial killing NK cells play an important role in target cell elimination (17).

## Conclusion

We report functional measurements allowing enumeration of cytotoxic cells and assessment of cytotoxic potential of individual cells in polyclonal populations of NK cells. The ability to monitor a single cell’s cytolytic capacity over time in a high throughput fashion is an advancement on standard techniques, such as isotope release assays (chromium/europium) or some flow-based methods, which perform bulk measurement of target cell death. While population measurements give a general overview, they fail to dissect how the sum of individual cellular responses contributes to the overall response. Flow-based CD107a release assays can yield single cell information on cellular degranulation but provides no information on, e.g., neither the number of target cells killed nor the temporal single cell history. On the flip side, the method presented here does require some technical know-how, is more laborious, and time consuming than population-based assays. These could be seen as limiting factors, e.g., clinical utility. However, this method offers a precise single cell tool, allowing small cell numbers to be observed over extended periods of time and seems especially useful when screening for rare events. We see these advantages as strong indicators of utility within hematopoietic stem cell transplantation where small, to date difficult to detect, populations of cells drive clinically beneficial graft-versus-leukemia effects or severe illness, such as GVHD. With a proof-of-concept, the clinical utility will outweigh the technical disadvantages and drive innovation and advancement toward an easier streamlined approach. Thus, we believe that the platform presented here, together with previously reported lab-on-a-chip tools, represent strong complements to flow- and population-based assays for evaluating NK cytotoxicity.

## Author Contributions

KG conducted a major part of the experiments, analyzed data, and wrote the article. LB performed some of the experiments, analyzed data, and developed image analysis software. K.Gust performed experiments. EF performed experiments and analyzed data. KO developed image analysis software. TF manufactured microchips and designed microchip holders. PO analyzed data. OM developed image analysis software. BV performed initial experiments. HB developed image analysis software. KK designed experiments. MU designed experiments. BÖ conceptualized experimental principle, designed the study, and wrote the paper.

## Conflict of Interest Statement

BÖ, MU, KK, BV and TF are inventors of a patent application related to the method.
