# Supplementary material for: Microchip Screening Platform for Single Cell Assessment of NK Cell Cytotoxicity
Source: Front Immunol. 2016 Apr 5;7:119. doi: 10.3389/fimmu.2016.00119 (PMC4820656; doi:10.3389/fimmu.2016.00119)
Supplement: Supplementary file 4 [file presentation_1.pdf]

SUPPLEMENTARY INFORMATION

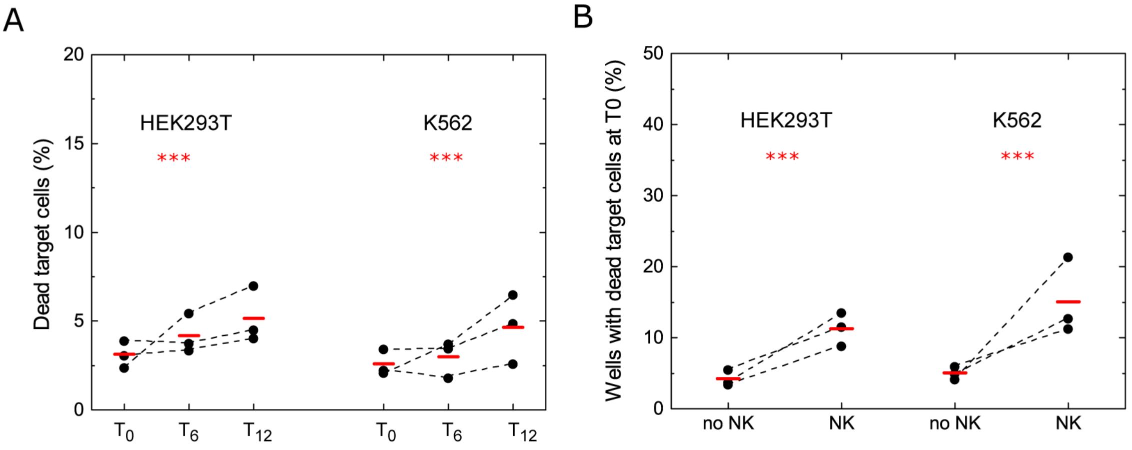

Figure S1. Background and spontaneous target cells death at the cellular level and in individual wells. (**A**). Fraction of dead HEK293T cells (left, n_{total}=3545) and K562 cells (right, n_{total}=7322) cells after seeding (T_0) and after 6 (T_{6h}) or 12 h (T_{12h}). Data is based on wells containing at least one target cell and no NK cells. Between 2-4% of the target cells were dead at seeding. While there were generally only small increases in the number of dead target cells during the first 6 h of the assay, more spontaneous death was observed during the second part. (**B**) Fraction of wells containing dead target cells at T_0 without (left, n_{293T}=2621 and n_{K562}=3895) or with (right, n_{293T}=1224 and n_{K562}=2216) NK cells. Based on statistical analysis of the total observations there was significantly (see red stars) more target cell death in wells containing NK cells reflecting that killing had started before the first set of images (corresponding to T_0) were acquired. Red bars indicate mean values.

Description of movies 1-3.

Resting NK cells were incubated with K562 cells in microwells and imaged every three minutes for twelve hours. Movies 1-3 show examples of NK cells killing 0, 2 or 8 target cells. Time stamps indicate what sections of the time-lapses that were used to make the movies. No killing events were observed outside the shown sequences.
